# The role of cuproptosis in the occurrence and development of gastric cancer

**DOI:** 10.3389/fphar.2025.1664200

**Published:** 2025-10-13

**Authors:** Ling Lu, Wenhui Yang, Yuning Gu, Longtao Jin, Zhaofeng Liang

**Affiliations:** ^1^ Child Healthcare Department, The Fourth Affiliated Hospital of Jiangsu University, Zhenjiang, Jiangsu, China; ^2^ Jiangsu Key Laboratory of Medical Science and Laboratory Medicine, School of Medicine, Jiangsu University, Zhenjiang, Jiangsu, China

**Keywords:** cuproptosis, gastric cancer, cellular death, molecular mechanisms, therapeutic strategies

## Abstract

Gastric cancer has emerged as a major global public health threat due to its high incidence and mortality rates. Despite advances in diagnostic and therapeutic approaches, treatment outcomes remain unsatisfactory with frequent disease recurrence and poor prognosis. This underscores the urgent need to develop novel therapeutic strategies against gastric cancer. Cuproptosis, a novel form of cellular death, has garnered increasing attention from researchers regarding its relationship with the onset and progression of gastric cancer. This article aims to explore the molecular mechanisms of cuproptosis, its functions in gastric cancer, and its potential therapeutic applications. We analyze the driving factors and defense mechanisms of cuproptosis, as well as how it influences the growth, metastasis, and drug resistance of gastric cancer cells. Furthermore, we discuss the interplay between cuproptosis and the gastric cancer microenvironment, and consider the possibilities of this mechanism in future clinical treatments.

## 1 Introduction

Gastric cancer is one of the leading causes of cancer-related mortality worldwide, characterized by a complex interplay of genetic, environmental, and microbial factors that contribute to its pathogenesis (Sundar et al., 2025; Ajani et al., 2025). Due to the multifaceted etiology, atypical symptoms, and complexity of the tumor microenvironment, early diagnosis of gastric cancer is particularly challenging, making prevention and treatment strategies complex (Gingrich et al., 2025; Li et al., 2025). *Helicobacter pylori* infection, along with dietary and genetic factors, can collectively cause chronic inflammation and lead to gastric cancer (Liu T. et al., 2025; Kesharwani et al., 2023). Recent studies emphasize the role of cell death mechanisms in cancer biology, highlighting new forms of regulated cell death that may have profound implications for the understanding and treatment of gastric cancer. Among these, cuproptosis represents a recently identified pathway gaining significant traction in cancer research (Yang et al., 2025; Guo et al., 2025).

Cuproptosis is characterized by the accumulation of copper ions within cells, leading to oxidative stress and subsequent cell death (Bhat et al., 2024; Chen et al., 2023). Copper overload, whether from external sources or internal metabolic issues, disrupts the tricarboxylic acid cycle. It impairs key enzymes and proteins, reduces energy production, damages mitochondrial function through oxidative stress, and can trigger cell death (Feng et al., 2024; Li et al., 2024; Lou et al., 2024). Understanding the mechanisms of cuproptosis is crucial, as it may provide insights into the metabolism of gastric cancer cells, which may provide new prevention and treatment strategies (Tsvetkov et al., 2022). The dysregulation of copper metabolism has been linked to various malignancies, including gastric cancer, suggesting that targeting this pathway could offer novel therapeutic strategies. Moreover, the complexity of gastric cancer is further compounded by its diverse histological subtypes (Liu X. et al., 2025; Kepil et al., 2019). Recent advancements in molecular profiling techniques have enabled researchers to delineate the genetic and epigenetic landscape of gastric tumors, facilitating the discovery of potential therapeutic targets (Lin et al., 2022). In summary, gastric cancer remains a formidable challenge due to its high mortality rate and the complexities of its pathogenesis. The emerging understanding of regulated cell death mechanisms, particularly cuproptosis, presents a promising avenue for future research and therapeutic intervention. By elucidating the role of copper metabolism in gastric cancer, researchers may identify novel strategies to enhance treatment efficacy and improve patient outcomes. As the field continues to evolve, it is imperative to integrate these insights into clinical practice to combat this deadly disease effectively.

This review provides new insights into the role of cuproptosis in gastric cancer by summarizing its molecular mechanisms and highlighting its dual impact on gastric cancer progression and treatment resistance. We focus on cuproptosis-related non-coding RNAs and their clinical utility as prognostic biomarkers. This review also explores the dynamic interplay between cuproptosis and the tumor immune microenvironment. Finally, emerging therapeutic strategies such as copper-based agents and combination therapies are discussed, offering a forward-looking perspective on targeting cuproptosis for improved clinical management of gastric cancer.

## 2 Definition and mechanism of cuproptosis

### 2.1 Concept and characteristics of cuproptosis

**FIGURE 1:**
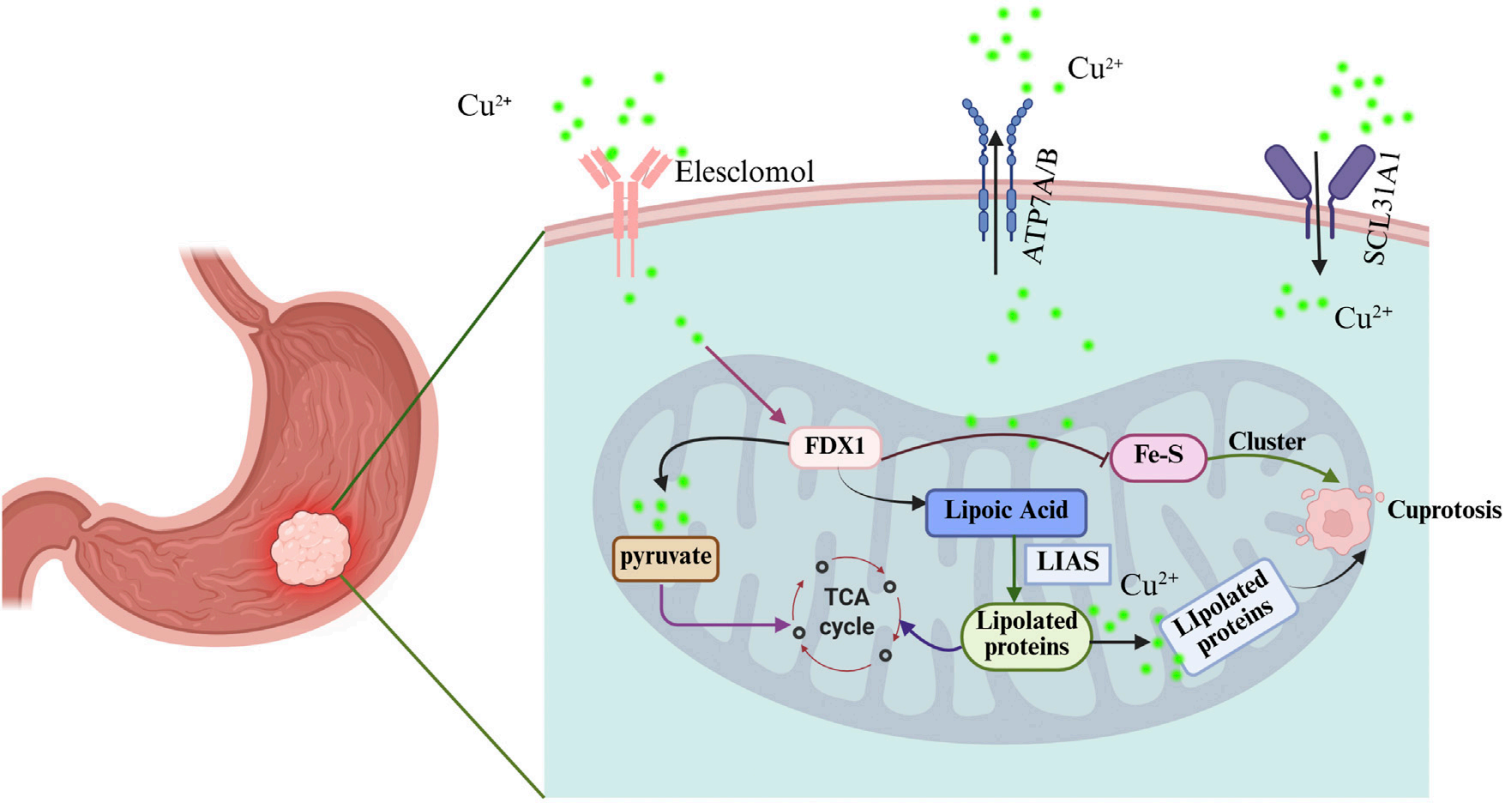
Schematic diagram of cuproptosis mechanism in gastric cancer. Created with BioRender.com.

Researchers recently characterized cuproptosis, a distinct form of regulated cell death separate from traditional apoptosis and necrosis (Chen et al., 2022; Wang Y. et al., 2022). It is characterized by its dependence on copper ions, which play a critical role in triggering this form of cell death. Recent studies have elucidated that cuproptosis is primarily induced by the accumulation of copper ions within the mitochondria, leading to mitochondrial dysfunction and the subsequent activation of specific cellular pathways that culminate in cell death (Feng et al., 2024; Tang et al., 2024). This process does not rely on the canonical apoptotic pathways typically mediated by caspases, but rather involves the disruption of mitochondrial metabolism and the dysregulation of iron-sulfur cluster proteins (Figure 1) (Wang et al., 2024; Zhang et al., 2023a; Zhou et al., 2023). The unique nature of cuproptosis positions it as a potential therapeutic target, especially in cancer treatment, where manipulating copper levels could selectively induce cell death in malignant cells while sparing normal cells.

### 2.2 Molecular basis and regulation of cuproptosis

The molecular underpinnings of cuproptosis involve several key proteins and regulatory mechanisms that respond to copper levels within the cell. Recent research has identified cuproptosis-related genes that are crucial for the execution of this form of cell death. These genes are implicated in various cellular processes, including mitochondrial function, oxidative stress response, and cell cycle regulation (Zhang et al., 2023b; Zhang S. et al., 2024). CDKN2A, MTF1, LIAS, GLS, FDX1 and CDKN2A have been highlighted as the regulators of cuproptosis, with its expression correlating with poor survival outcomes in cancers (Zhang et al., 2023a; Wu et al., 2025; Zuo et al., 2025; Zhang R. et al., 2024). Moreover, copper metabolism and cuproptosis-related genes interact in ways that may affect both cancer development and treatment response. Understanding this relationship could open new paths for therapy.

#### 2.2.1 Role and sources of copper ions

Copper ions play a dual role in biological systems, acting as essential cofactors in enzymatic reactions while also posing a risk for toxicity when present in excess. The sources of copper in the human body include dietary intake, which is vital for maintaining copper homeostasis, and cellular uptake mechanisms that regulate copper levels within tissues. Dysregulation of copper homeostasis has been linked to various diseases, including cancer, neurodegenerative disorders, and cardiovascular diseases (Zhang S. et al., 2024; Xu et al., 2024). In the context of cuproptosis, excess copper can lead to the activation of cell death pathways through mitochondrial stress and the disruption of iron-sulfur clusters (Wu et al., 2023; Chen T. et al., 2024). Understanding the sources and roles of copper ions is crucial for developing strategies to manipulate cuproptosis for therapeutic benefits, particularly in targeting cancer cells that exhibit altered copper metabolism.

### 2.3 Cuproptosis in gastric cancer cells

#### 2.3.1 Sensitivity of gastric cancer cells to cuproptosis

Gastric cancer cells exhibit a notable sensitivity to cuproptosis. This type of cell death is characterized by its dependence on mitochondrial respiration and is triggered by copper accumulation within cells. Research indicated that gastric cancer cells respond significantly to elevated copper levels, which can induce apoptosis through various mechanisms, including the disruption of mitochondrial function and the generation of ROS (Huang et al., 2024). New copper-based compounds, like a specific Schiff base copper complex, have been shown to effectively block stomach cancer growth. They work better than cisplatin in both lab-grown cancer cells and mouse studies, requiring lower doses to achieve the same effect (Xia et al., 2019). These experimental evidences provide a theoretical basis for us to understand the role of cuproptosis in cancers such as gastric cancer. The underlying mechanisms involve the activation of apoptotic pathways and cell cycle arrest, highlighting the potential of copper as a therapeutic agent against gastric cancer.

#### 2.3.2 Impact of cuproptosis on gastric cancer cell proliferation

The induction of cuproptosis has profound implications for the proliferation of gastric cancer cells. Evidence suggests that cuproptosis can lead to significant growth inhibition in various gastric cancer cell lines. KYNU promoted the proliferation of gastric cancer cells by mediating cuproptosis resistance confirmed in cell models and clinical samples (Liu Y. et al., 2025). For instance, the Schiff base copper coordinated compound not only inhibited cell proliferation but also induced apoptosis via multiple signaling pathways, including the inhibition of NF-κB and the production of ROS in cells and mouse models (Xia et al., 2019). The expression of cuproptosis-related genes has been correlated with clinical outcomes in gastric cancer, indicating that these genes may serve as potential prognostic markers (Zhao B. et al., 2023). Research has shown that ITGB1 regulates tumor metabolism and copper deposition in diffuse type gastric cancer, affects cell proliferation and motility, and promotes gastric cancer in cell models and clinical samples (Zhu et al., 2023). Copper’s ability to modulate key processes like glycolysis and autophagy underscores its role in suppressing tumor growth. This makes it a promising target for developing new therapies that exploit its cytotoxic properties against gastric cancer.

#### 2.3.2 Role of cuproptosis in the migration and invasion of gastric cancer cells

Cuproptosis also significantly influences the migratory and invasive capabilities of gastric cancer cells. The mechanisms through which cuproptosis affects these processes are multifaceted and involve alterations in the tumor microenvironment and immune response. For example, study has shown that the expression of cuproptosis-related genes is associated with the infiltration of various immune cells, which can modulate tumor behavior and metastatic potential, as evidenced through database and clinical sample analysis (Wang L. et al., 2023). KYNU promoted the invasion, and metastasis of gastric cancer cells by mediating cuproptosis resistance in cell models and clinical samples (Liu Y. et al., 2025). Wu et al. discovered that MTF1 is a key gene in cuproptosis and was found to affect apoptosis and invasion of cancer cell lines (Wu et al., 2025). Studies by Zhou et al. show that targeting PTBP3-mediated COX11 splicing triggers cuproptosis, which inhibits gastric cancer peritoneal metastasis across cell, animal, and clinical models (Zhou et al., 2025). PTBP3-mediated exon 4 skipping in COX11 pre-mRNA is critical for tumor cell survival and progression in gastric cancer peritoneal metastasis, providing a potential therapeutic strategy targeting copper metabolism. Additionally, cuproptosis has been linked to changes in the expression of proteins involved in cell adhesion and migration in cell models and clinical samples (Peng et al., 2024).

These studies suggest that targeting copper levels or cuproptosis pathways could provide novel strategies to inhibit gastric cancer progression and enhance its sensitivity to cuproptosis (Table 1), thereby improving patient outcomes. The integration of cuproptosis-related biomarkers into clinical practice may also enhance the precision of therapeutic approaches, tailoring interventions based on individual tumor characteristics and responses to copper modulation.

**TABLE 1:** Role of cuproptosis in the proliferation, migration and invasion of gastric cancer cells.

Key molecules	The role in gastric cancer	Possible targets	Experimental model	References
KYNU	Promoted cell proliferation, invasion, metastasis, and cuproptosis resistance	LIAS	Cell models and clinical samples	Liu et al. (2025c)
Schiff base copper coordinated compound	Inhibited cell proliferation	NF-κB, ROS production	Cells and mouse models	Xia et al. (2019)
ITGB1	Regulated tumor metabolism and cuproptosis	ROCK1, PKACA/PRKACA, AKT1, FDX1, DLAT, and DLST	Cell models and clinical samples	Zhu et al. (2023)
LncRNA AC129926.1	Promoted migration and proliferation	—	Database and clinical sample analysis	Wang et al. (2023a)
CDKN2A	Regulated the invasion and apoptosis	MTF1	Cell models	Wu et al. (2025)
PTBP3	Induced cuproptosis and Inhibited peritoneal metastasis	COX11	Cell/animal models and clinical samples	Zhou et al. (2025)
DLAT	Affected the invasion and migration	BTN2A1	Cell models and clinical samples	Peng et al. (2024)

### 2.4 Drivers of cuproptosis

#### 2.4.1 Oxidative stress and cuproptosis

Cuproptosis is characterized by the accumulation of copper ions, which induces oxidative stress by overwhelming cellular antioxidant defenses (Feng et al., 2024; Zhang Y. et al., 2024). Elevated copper levels lead to the generation of ROS, resulting in cellular damage and ultimately apoptosis. The interaction of copper with TCA cycle triggers the aggregation of these proteins and the loss of iron-sulfur cluster proteins, contributing to proteotoxic stress and cell death (Lou et al., 2024; Xu et al., 2023). Oxidative stress is a key driver in neurodegenerative pathogenesis, and copper dysregulation further exacerbates cognitive decline by inducing oxidative damage and mitochondrial dysfunction. Thus, the relationship between oxidative stress and cuproptosis highlights the critical balance required for copper homeostasis in maintaining cellular integrity and function (Lou et al., 2024; Li X. et al., 2023). Oxidative stress and cuproptosis play important roles in the mechanisms, prognosis, and treatment of tumors, including gastric cancer (Feng et al., 2024; Zhang Y. et al., 2024; Vo et al., 2024). Some researchers have begun to design treatment strategies based on cuproptosis and oxidative stress from a theoretical perspective (Xu et al., 2023; Wen et al., 2025). Fully understand cuproptosis, especially through its interaction with copper induced oxidative stress to exert its anti-cancer activity, thus providing new ideas for future treatment methods targeting cell death patterns.

#### 2.4.2 Impact of redox imbalance

The delicate balance of redox homeostasis is vital for cellular health, and disruptions in this equilibrium can lead to various forms of regulated cell death, including cuproptosis. Copper, with its dual role as a pro-oxidant and cofactor for antioxidant enzymes, can induce redox imbalance when present in excess. This imbalance is particularly detrimental in cancer cells, which often exhibit altered metabolic pathways favoring glycolysis over oxidative phosphorylation. The accumulation of copper ions in these cells can lead to excessive ROS production, triggering cell death through cuproptosis. Furthermore, activation of pathways such as Wnt/β-catenin signaling confers resistance to cuproptosis by promoting copper efflux, which maintains low intracellular copper levels and protects cancer cells. Thus, understanding the interplay between copper metabolism, redox balance, and cell death mechanisms is essential for developing targeted therapies for conditions associated with copper dysregulation, particularly in oncology using cell and animal models (Liu et al., 2024; Cun et al., 2025). Zhao et al. developed metal-phenolic network nanoparticles that disrupt redox homeostasis to synergize chemo/chemodynamic therapy through concurrent induction of apoptosis and cuproptosis *in vitro* and *in vivo* models (Zhao F. et al., 2023).

#### 2.4.3 Regulation of cuproptosis by non-coding RNA

Non coding RNAs such as miRNAs and lncRNAs have become key regulators of gene expression and cellular processes, including those involved in copper metabolism and copper metabolism. Specific non-coding RNAs can not only regulate the expression of copper transporters and cuproptosis-related proteins but also serve as prognostic biomarkers and therapeutic targets for gastric cancer.

MiRNAs such as miR-21 and miR-155 have been shown to influence the expression of genes related to oxidative stress response and copper homeostasis through *in vivo* and *in vitro* models, as well as bioinformatics analysis (Qian et al., 2023; Jiang F. et al., 2023). Dysregulation of these miRNAs can lead to altered copper levels within cells, promoting either cytotoxicity or survival. Moreover, the interplay between miRNAs and copper-related genes highlights a complex regulatory network that can affect cellular responses to copper overload. This regulatory mechanism is particularly relevant in cancer, where miRNA profiles can influence the sensitivity of tumor cells to copper-induced death, presenting potential avenues for therapeutic intervention. By targeting specific miRNAs, it may be possible to enhance the efficacy of copper-based therapies in cancer treatment (Graham et al., 2023; Wang J. et al., 2023).

Using multivariate Cox analysis techniques, Yin et al. developed a feature based on four lncRNAs related to cuproptosis. Classify patients into high-risk and low-risk groups based on the likelihood of adverse outcomes, providing a basis for the diagnosis and treatment of gastric cancer patients (Yin et al., 2023). Based on bioinformatics analysis, Ding et al. elucidated a ceRNA mechanism in gastric cancer where lncMALAT1 binds directly to miR-328-3p, consequently alleviating its repression and upregulating the key cuproptosis gene FDX1 (Ding et al., 2023). This MALAT1/miR-328-3p/FDX1 axis was validated through dual-luciferase assays, RT-qPCR, and rescue experiments, confirming its functional role in modulating cuproptosis. Tu et al. discovered six cuproptosis associated lncRNAs as prognostic factors for gastric cancer, which may be useful biomarkers for stratifying gastric cancer risk, evaluating potential immunotherapy and assessing treatment sensitivity (Tu et al., 2022). Qu and colleagues pinpointed four cuproptosis associated lncRNAs linked to gastric cancer patient outcomes (AC016394.2, NUTM2A-AS1, OIP5-AS1, and LIMS1-AS1) and developed a prognostic risk model. The model’s risk score showed strong ties to key tumor microenvironment features, including tumor mutational burden, immune subtypes, and immune cell infiltration (Qu et al., 2025). Cuproptosis-associated lncRNAs serve as the very precise prognostic biomarkers in gastric cancer, effectively discriminating immunologically cold and hot tumor subtypes, thereby provide a basis for the precision medicine of gastric cancer (Huang et al., 2022). Collectively, these studies demonstrated that cuproptosis-related lncRNAs not only serve as prognostic biomarkers but also actively regulate cuproptosis and shape the immune tumor microenvironment. Integrating these lncRNAs into molecular subtyping systems and therapeutic strategies of gastric cancer offers a promising strategy for advancing precision medicine in gastric cancer.

Non-coding RNAs, notably miRNAs and lncRNAs, emerge as pivotal modulators of cuproptosis in gastric cancer. They orchestrate copper homeostasis by targeting transporters and death pathways, while their dysregulation directly impacts gastric cancer cell susceptibility to cuproptosis. These findings collectively point to non-coding RNAs as both mechanistic regulators and therapeutic targets for prognostication and therapy. However, some non-coding RNAs are currently only used as predictive markers, and their exact roles, mechanisms, and clinical value still require a lot of experimental research and clinical cohort studies to verify.

### 2.5 Interaction between cuproptosis and gastric cancer microenvironment

Recent studies have shown that abnormal regulation of copper metabolism is closely associated with malignant progression in several types of cancer, including gastric cancer (He et al., 2023; Chen Y. et al., 2024). Understanding the relationship between copper metabolism and cell death, especially the mechanism of action in the tumor microenvironment, may provide new breakthroughs for cancer treatment. Cuproptosis is a key driver in remodeling the gastric tumor microenvironment, primarily affecting immune evasion, stromal activation, and angiogenesis. Copper accumulation modulates tumor-infiltrating immune cells by regulating cuproptosis-related genes like FDX1 and LIAS, which influence T-cell activity and macrophage polarization to alter immune surveillance. Tumor-associated fibroblasts also respond to copper stress by secreting more pro-tumorigenic factors. This may drive extracellular matrix remodeling and foster therapy resistance. Angiogenesis represents another critical axis where copper homeostasis may exert influence, though mechanistic insights remain limited, especially regarding endothelial cell responses to cuproptosis signals. Importantly, current studies have not fully elucidated the spatial and temporal dynamics of these interactions within human gastric tumors.

#### 2.5.1 Role of tumor-associated fibroblasts

Tumor-associated fibroblasts (TAFs) play a pivotal role in the gastric cancer microenvironment, significantly influencing tumor progression and response to therapies. Recent studies indicate that TAFs can modulate the effects of cuproptosis, by altering the extracellular matrix and cytokine profiles within the tumor niche. The interaction between copper levels and TAFs may create a unique microenvironment that facilitates tumor growth and metastasis. Elevated copper levels have been shown to affect the behavior of TAFs, promoting a more aggressive tumor phenotype. Li et al. found a close association between arecoline, cuproptosis, and TAFs, which may play an important role in the metastasis of Oral Squamous Cell Carcinomas (Li et al., 2022). Cuproptosis-related lncRNAs exhibit a significant association with TAFs in osteosarcoma, offering potential value for prognostic evaluation and targeted treatment of osteosarcoma patients (Wang X. et al., 2023). TAFs promoted oral squamous cell carcinoma progression by modulating cuproptosis through exosome-mediated transfer of miR-148b-3p, which targets ATP7A (Zhang et al., 2025). TAFs can secrete factors that enhance angiogenesis and immune evasion, thereby supporting tumor survival and growth in the presence of copper-induced stress. By altering TAFs, cuproptosis can reshape the gastric tumor immune landscape into a more immunosuppressive state, thereby complicating treatment. Developing therapies that disrupt the role of TAFs in gastric cancer requires a deeper understanding of their involvement in copper metabolism and cuproptosis (Zhao B. et al., 2023; Li et al., 2023b). Recent research has highlighted that copper levels can influence the infiltration and activation of various immune cell types, including T cells, macrophages, and dendritic cells. Elevated copper concentrations can induce cuproptosis, leading to the release of damage-associated molecular patterns that activate immune responses. For example, cuproptosis can promote immunogenic cell death, enhance the presentation of tumor antigens and stimulating T cell activation.

#### 2.5.2 Role of immune cells in cuproptosis

Immune cells are integral to the tumor microenvironment and play a complex role in the process of cuproptosis in gastric cancer. Recent research has highlighted that copper levels influence the activation of various immune cell types, including T cells, macrophages, and dendritic cells. High copper levels trigger cuproptosis, which sends out signals that activate the immune system. For example, cuproptosis can promote immunogenic cell death, enhance the presentation of tumor antigens and stimulating T cell activation. The immune response, however, is a double-edged sword. Some cells rally to fight the tumor, while others shield it by suppressing the local environment. The balance between these opposing effects is crucial for determining the overall efficacy of immunotherapies in gastric cancer. Understanding the connection between immune cell and cuproptosis could provide insights into novel therapeutic strategies aimed at enhancing antitumor immunity while overcoming the immunosuppressive mechanisms employed by tumors (Zhao B. et al., 2023; Li et al., 2023b). Nie et al. confirmed through analysis of immune therapy response that gastric cancer patients with high cuprotosisScore can better benefit from immune therapy and have better sensitivity to chemotherapy drugs (Nie et al., 2023). Copper death related genes such as LIAS, GLS, and CDKN2A can regulate the function of immune cells, affect the occurrence and development of gastric cancer, and serve as biomarkers for gastric cancer patients (Zhang R. et al., 2024). In gastric cancer patients, lower cuproptosis-related human copper proteome scores were associated with an enhanced anti-tumor immune microenvironment (Tang et al., 2022). Moreover, these patients exhibited higher tumor mutational burden/microsatellite instability levels and demonstrated enhanced response to immunotherapy. In summary, the interaction between cuproptosis and immune evasion in gastric cancer involves a dynamic and context-dependent balance. On one hand, cuproptosis can enhance antitumor immunity by promoting immunogenic cell death and activating T cells. On the other hand, it may also contribute to an immunosuppressive microenvironment through altered immune cell functions and stromal remodeling. Fully elucidating this dual role will be crucial for developing effective strategies that leverage cuproptosis to improve immunotherapy outcomes in gastric cancer.

#### 2.5.3 Impact of cuproptosis on tumor microenvironment

Analysis of 1,401 gastric cancer patients revealed cuproptosis has significant implications for the tumor microenvironment in gastric cancer (Wang X. X. et al., 2023). By rewiring the tumor microenvironment, cuproptosis can fundamentally change tumor progression and treatment outcomes. When cuproptosis is triggered, it results in the death of cancer cells, which can subsequently release inflammatory mediators that attract immune cells to the tumor site. This immune infiltration may enhance anti-tumor response, but if the immune response is dysregulated, it may also promote tumor growth. Additionally, the metabolic changes associated with cuproptosis can influence the availability of nutrients and oxygen within the tumor, further modulating the behavior of surrounding cells, including fibroblasts and immune cells. The connection between copper, cuproptosis, and the tumor microenvironment points to new ways of treating gastric cancer (Zhao B. et al., 2023; Li et al., 2023b). Nie et al. found that compared with patients with high cuprotosisscore, patients with low cuprotosisscore had poorer prognosis, enhanced tumor microenvironment infiltration, higher TNM staging, and stronger matrix activation (Nie et al., 2023). Wang’s research conducted a comprehensive analysis of cuproptosis related genes in gastric cancer, indicating the important regulatory roles in the gastric cancer microenvironment, and prognosis. This may provide new insights for doctors to predict the prognosis of gastric cancer and develop more effective and personalized treatment strategies patients tissues (Wang J. et al., 2022). SERPINE1 is highly expressed in gastric cancer patients’ tissues and is associated with poor prognosis. SERPINE1 may regulate cuproptosis and immune microenvironment through a series of pathways (Feng et al., 2023). Dong and Li et al. reported that cuproptosis related genes can affect the tumor microenvironment and prognosis of gastric cancer patients (Dong et al., 2022; Li et al., 2023c). Based on these information, personalized tumor treatment strategies can be provided for gastric cancer patients. Overall, these findings underscore the critical role of cuproptosis in dynamically reshaping the gastric tumor microenvironment, influencing both immunoreactivity and stromal remodeling, thereby offering new avenues for prognostic assessment and personalized therapeutic intervention.

### 2.6 The prospects of cuproptosis in gastric cancer treatment

Gastric cancer is one of the most common malignant tumors of the digestive system. This chapter primarily explores the role of cuproptosis in gastric cancer treatment, providing new insights for predicting disease progression and selecting therapeutic strategies. It also identifies potential therapeutic targets related to cuproptosis in gastric cancer, laying the foundation for future drug development and treatment optimization based on these targets and research findings. Recent studies have shown that cuproptosis differs significantly from previously understood mechanisms of cell death, and cuproptosis regulation in gastric cancer cells may be a novel and clinically promising therapeutic strategy (Table 2).

**TABLE 2:** The prospects of cuproptosis in gastric cancer treatment.

Key molecules/pathway	Target	Roles	Experimental model	References
METTL16	FDX1/NF-κB	Induced cuproptosis and improve the therapeutic efficacy of the copper ionophore- elesclomol	Cells and mouse models	Sun et al. (2023)
lSchiff base copper coordinated compound	NF-κB, ROS production	Inhibited cell proliferation and overcome the side-effect for clinical application	Cells and mouse models	Xia et al. (2019)
ENTPD3, PDZD4, CNN1, GTPBP4, FPGS, UTP25, CENPW and FAM111A	—	Early diagnosis, tumor classification, drug screening	Clinical samples	Jiang et al. (2023b)
VCAN-AS1, HAGLR, CDKN2B-AS1 and AL359704.2	BEZ235/PI3K/mTOR	Risk prediction; Immune function and drug sensitivity assessment	Database and clinical sample analysis	Yin et al. (2023)
CDKN2A	MTF1	Predicted the prognosis of gastric cancer patients	Clinical samples	Wu et al. (2025)
CDKN2A, DLD, GLS, MTF1, DLAT, FDX1、PDHA1, PDHB	—	Predicted the clinical stage, prognosis and overall survival	Database and clinical sample analysis	Ding et al. (2023)
FDX1, LIAS, and MTF1	—	Prognostic markers and immune targeted therapy targets	Database and clinical sample analysis	Yan et al. (2023)
AC008915.2, AC011005.4, AC023511.1, AC139792.1, AL355312.2, LINC01094 and LINC02476	—	Prognosis and tumor immune markers	Cells and mouse models	Zhao et al. (2023a)
Disulfiram	S6K1, c-Myc	Inhibited glycolysis, proliferation, and tumor growth	Cells and mouse models	Du et al. (2022)
DLAT, GCSH, PDHA1, FDX1, LIPT1, ATP7B, DLD and SLC31A1	m6A-, and m7G-related genes, INF	Predicted the prognosis	Database and clinical sample analysis	He et al. (2023)
LINC01150	CD209 and HAVCR2	Prognosis and tumor immune markers	Clinical samples	Feng et al. (2022)
SiO2@Cu2-xSe nanospheres	—	NIR mediated treatment	Cell and animal models	Zhang et al. (2020)
FDX1	CDKN2A	Predicted survival and overcomed drug resistance	Database and clinical sample analysis	Zuo et al. (2025)
FDX1	—	Potential diagnostic biomarker and therapeutic target	Database and clinical sample analysis	Xie et al. (2024)
DBT, MTF1, ATP7A, ATP7B, SLC31A1, GCSH, LIAS, DLAT, FDX1, DLD, and PDHA1	—	Characterized clinical features and molecular landscape	Database/clinical sample analysis and cell models	Chong et al. (2024)

#### 2.6.1 As a new target for antitumor therapy

Cuproptosis, or cuproptosis, is an emerging mechanism of programmed cell death that has garnered attention in cancer research, particularly in gastric cancer. This process is characterized by the accumulation of copper ions, leading to the aggregation of lipoylated proteins and subsequent cellular stress, ultimately resulting in cell death. Cuproptosis caused by excessive copper concentration is urgently being developed as a potential cancer treatment method. Sun et al. found a significant increase in copper content in gastric cancer, especially in malignant tumors *in vitro* and *in vivo* (Sun et al., 2023). METTL16 is an atypical methyltransferase and a key mediator of cuproptosis. Elevated acetylation of METTL16 significantly enhances the therapeutic effect of copper ionophore-elesclomol.

Recent studies have highlighted the significance of copper homeostasis in cancer progression, indicating that both excess and deficiency of copper can influence tumor behavior. The potential of targeting cuproptosis as a therapeutic strategy is particularly promising, as it may provide a novel approach to induce cell death in cancer cells that exhibit resistance to conventional therapies. For instance, manipulating copper levels in gastric cancer cells has shown to enhance the effectiveness of existing treatments, presenting cuproptosis as a viable target for novel anticancer drugs aimed at exploiting this unique form of cell death (Huang et al., 2024; Xia et al., 2019). Jiang et al. reported that the candidate drug dasatinib may effectively treat gastric cancer by affecting the expression of cuproptosis marker genes in gastric cancer tissues (Jiang L. et al., 2023). It is crucial to study the prognostic importance of cuproptosis related lncRNAs for the prognosis and treatment response of gastric cancer (Yin et al., 2023). Wu et al. reported that overall mutations in cuproptosis genes, changes in PDHB copy number, methylation of CDKN2A, alternative splicing, and APA alterations all affect the prognosis of gastric cancer patients (Wu et al., 2025). Ding et al.'s study showed a significant correlation between FDX1 expression and immune cell infiltration, tumor mutation burden score, microsatellite instability score and drug sensitivity in gastric cancer based on bioinformatics analysis (Ding et al., 2023). Yan et al. found through screening that FDX1, LIAS, and MTF1 can serve as potential prognostic biomarkers for gastric cancer patients and provide new targets for immune targeted therapy based on bioinformatics analysis (Yan et al., 2023).

Cuproptosis, driven by copper-induced lipoylated protein aggregation, represents a promising therapeutic target in gastric cancer, particularly given the elevated copper levels observed in malignant tumors. Disrupted copper homeostasis critically influences gastric cancer progression, making cuproptosis induction a viable strategy against therapy-resistant cells, potentially augmenting existing treatments.

#### 2.6.2 Development and clinical application of copper-based drugs

The exploration of copper-based compounds as therapeutic agents in gastric cancer has gained momentum, with several studies investigating their antitumor properties. One notable development is the synthesis of novel copper complexes that exhibit superior cytotoxicity against gastric cancer cells compared to traditional chemotherapeutics. For example, a newly synthesized Schiff base copper compound demonstrated significant inhibition of gastric cancer cell proliferation and induced apoptosis through multiple pathways in the cell model (Xia et al., 2019). Furthermore, copper chelators and ionophores have been shown to enhance the efficacy of existing chemotherapeutics by increasing intracellular copper levels, thereby promoting cuproptosis in cancer cells. The clinical implications of these findings suggest that copper-based therapies could be integrated into treatment regimens for gastric cancer, potentially improving patient outcomes *in vitro* and *in vivo* (Zhao B. et al., 2023; Du et al., 2022). He et al. found a significant correlation between multiple cuprotosis related genes and the prognosis of gastric cancer patients based on database and clinical sample analysis (He et al., 2023).

#### 2.6.3 Combined application of cuproptosis with other treatment strategies

The integration of cuproptosis-inducing agents with other therapeutic modalities presents a promising avenue for enhancing the efficacy of gastric cancer treatment. Recent studies have demonstrated that combining copper-based therapies with immunotherapy or targeted therapies can synergistically enhance antitumor effects. The co-administration of disulfiram, a drug known for its copper-chelating properties, has been shown to significantly enhance the therapeutic efficacy of immune checkpoint inhibitors by promoting cuproptosis in gastric cancer patients (Feng et al., 2022). Additionally, the use of copper nanoparticles in conjunction with photothermal therapy has shown potential in targeting gastric cancer cells effectively, leveraging the unique properties of copper to induce localized heating and subsequent cell death *in vitro* and *in vivo* (Zhang et al., 2020). These combined strategies not only aim to maximize the therapeutic impact on gastric cancer cells but also to overcome challenges associated with drug resistance, thereby paving the way for more effective treatment protocols (Huang et al., 2024; Xie et al., 2024). Zou et al. identified cuproptosis related genes were significantly upregulated, which is associated with improved prognosis and immune cell infiltration patterns in gastric cancer patients (Zuo et al., 2025). The prognostic model combining FDX1, PDHA1, and LIAS expression has the potential to classify gastric cancer patients into different risk categories as biomarkers for personalized treatment strategies. Use the Copper Dropping Characteristic Risk Score (CSRS) scheme to assess the prognosis and risk of individual gastric cancer (Chong et al., 2024). The characteristic of CSRS low scoring patients is delayed survival time. Further analysis indicates that low CSRS scores are also associated with a greater burden of tumor mutations and a higher rate of significant gene mutations in GC. In addition, CSRS can be used to screen potential drugs for the treatment of different types of gastric cancer.

While cuproptosis presents a compelling novel therapeutic axis in gastric cancer. Future research must delineate the precise molecular mechanisms governing copper sensitivity and cuproptosis execution within the tumor microenvironment. Translating promising *in vitro* findings on copper-based drugs into clinically viable and targeted delivery systems is paramount, alongside defining therapeutic windows that avoid systemic cuproptosis. Rigorous validation of proposed prognostic biomarkers and multi-gene signatures like the CSRS across diverse patient cohorts is essential for their clinical utility in risk stratification and personalization.

To optimize treatments that combine cuproptosis inducers with immunotherapy or other therapies, we need concerted preclinical and clinical efforts to overcome resistance and improve efficacy. Understanding the interplay between cuproptosis, the immune landscape, and genomic instability will be fundamental for developing these integrated strategies.

## 3 Future perspectives and challenges

The translation of cuproptosis induction into clinically actionable strategies for gastric cancer faces several critical challenges that must be addressed in future research. Systemic cuproptosis remains a major concern, as excessive copper accumulation can lead to nonspecific tissue damage and neurological impairment. This underscores the need for strategies that enhance tumor-selective copper delivery while minimizing off-target effects. A primary obstacle is the current lack of standardized and validated assays for detecting cuproptosis in human tissue samples. Developing reliable biomarkers, such as immunohistochemical or functional tests that quantify lipoylated protein aggregation or iron-sulfur cluster loss, is essential. These tools will allow us to objectively evaluate cuproptosis in clinical samples and correlate it with treatment response. Equally important is the identification of robust predictive biomarkers to guide patient selection. Validating biomarkers like FDX1, LIAS, or related non-coding RNAs could help identify gastric cancer subtypes that are vulnerable to copper-mediated cell death, thereby enabling more personalized therapeutic approaches. Furthermore, rational combination strategies represent a promising avenue to enhance efficacy and overcome resistance. Given the immunomodulatory potential of cuproptosis, combining copper ionophores such as elesclomol with immune checkpoint inhibitors warrants systematic investigation. Additionally, synergies with conventional chemotherapy, targeted agents, or radiotherapy should be explored in both preclinical and clinical settings. Finally, a deeper understanding of the spatiotemporal dynamics between cuproptosis and the tumor microenvironment is needed. Advanced techniques like spatial transcriptomics and multiplexed imaging could elucidate how cuproptosis influences immune cell infiltration, stromal activation, and metabolic crosstalk, ultimately informing more effective combination regimens. Addressing these gaps will be crucial to harnessing cuproptosis as a clinically relevant therapeutic strategy in gastric cancer.

## 4 Conclusion

Cuproptosis has emerged as a significant player in the pathogenesis and progression of gastric cancer, shedding light on novel therapeutic avenues. The intricate molecular mechanisms underlying cuproptosis, alongside its biological functions, suggest that manipulating copper levels may offer a promising strategy for gastric cancer treatment. However, balancing the diverse perspectives and findings from various studies remains crucial for translating these insights into clinical applications. The current body of research highlights both the potential and the challenges of cuproptosis in gastric cancer. While some studies demonstrate that copper accumulation can induce apoptosis in cancer cells, others indicate a more complex relationship where copper may also confer survival advantages under specific circumstances. To harmonize these differing viewpoints, future research must focus on delineating the conditions that dictate copper’s role in gastric cancer. For instance, further investigations could explore the interplay between copper levels and other metabolic pathways, as well as the tumor microenvironment’s influence on cuproptosis. Additionally, the development of targeted therapies that modulate copper metabolism could pave the way toward more personalized treatment regimens. In conclusion, while the exploration of cuproptosis in gastric cancer presents exciting opportunities, it is imperative to approach the subject with a critical lens. A comprehensive understanding of the molecular underpinnings, combined with careful consideration of the clinical implications, will be essential in overcoming the existing hurdles. By fostering interdisciplinary research efforts and collaborative studies, we can unlock the full potential of cuproptosis as a therapeutic strategy for gastric cancer, ultimately improving patient outcomes.

## 5 Limitations

It should be acknowledged that current evidence regarding cuproptosis in gastric cancer predominantly originates from cell-based experiments, animal models, and retrospective bioinformatic analyses. While these studies provide crucial mechanistic insights and suggest therapeutic potential, they have not yet been substantiated by large-scale prospective clinical trials. Therefore, the implications discussed in this review should be interpreted as hypothesis-generating rather than as confirmed clinical evidence. Further translational research and rigorously designed clinical studies are needed to validate the role of cuproptosis in gastric cancer and its applicability to human patients.
